# A Frameshift Mutation in Golden Retriever Dogs with Progressive Retinal Atrophy Endorses *SLC4A3* as a Candidate Gene for Human Retinal Degenerations

**DOI:** 10.1371/journal.pone.0021452

**Published:** 2011-06-27

**Authors:** Louise M. Downs, Berit Wallin-Håkansson, Mike Boursnell, Stefan Marklund, Åke Hedhammar, Katarina Truvé, Louise Hübinette, Kerstin Lindblad-Toh, Tomas Bergström, Cathryn S. Mellersh

**Affiliations:** 1 Canine Genetics, Animal Health Trust, Newmarket, United Kingdom; 2 The Swedish Kennel Club (SKK), Stockholm, Sweden; 3 Department of Clinical Sciences, Swedish University of Agricultural Sciences (SLU), Uppsala, Sweden; 4 Department of Animal Breeding and Genetics, Swedish University of Agricultural Sciences (SLU), Uppsala, Sweden; 5 Science for Life Laboratory, Department of Medical Biochemistry and Microbiology, Uppsala University, Uppsala, Sweden; 6 The Broad Institute of Harvard University and the Massachusetts Institute of Technology, Cambridge, Massachusetts, United States of America; Ohio State University Medical Center, United States of America

## Abstract

Progressive retinal atrophy (PRA) in dogs, the canine equivalent of retinitis pigmentosa (RP) in humans, is characterised by vision loss due to degeneration of the photoreceptor cells in the retina, eventually leading to complete blindness. It affects more than 100 dog breeds, and is caused by numerous mutations. RP affects 1 in 4000 people in the Western world and 70% of causal mutations remain unknown. Canine diseases are natural models for the study of human diseases and are becoming increasingly useful for the development of therapies in humans. One variant, *prcd*-PRA, only accounts for a small proportion of PRA cases in the Golden Retriever (GR) breed. Using genome-wide association with 27 cases and 19 controls we identified a novel PRA locus on CFA37 (p_raw_ = 1.94×10^−10^, p_genome_ = 1.0×10^−5^), where a 644 kb region was homozygous within cases. A frameshift mutation was identified in a solute carrier anion exchanger gene (*SLC4A3*) located within this region. This variant was present in 56% of PRA cases and 87% of obligate carriers, and displayed a recessive mode of inheritance with full penetrance within those lineages in which it segregated. Allele frequencies are approximately 4% in the UK, 6% in Sweden and 2% in France, but the variant has not been found in GRs from the US. A large proportion of cases (approximately 44%) remain unexplained, indicating that PRA in this breed is genetically heterogeneous and caused by at least three mutations. *SLC4A3* is important for retinal function and has not previously been associated with spontaneously occurring retinal degenerations in any other species, including humans.

## Introduction

Retinitis pigmentosa (RP) is the collective name for a group of inherited human retinal disorders that leads to progressive loss of vision in approximately 1 in 4000 people [1], [2], [3]. Rod photoreceptor cells are predominantly affected and therefore clinical symptoms typically include night blindness and loss of peripheral vision. With disease progression the cones also degenerate resulting in central vision loss and eventually complete blindness is possible. To date, 167 genes have been shown to cause a wide spectrum of retinal disease, including RP (RetNet; http://www.sph.uth.tmc.edu/retnet/). Mutations in these genes currently only account for approximately 30% of recessive RP cases. [4].

In animals inherited and progressive retinal diseases are commonly referred to as progressive retinal atrophy (PRA) and as in humans PRA is characterised by progressive retinal degeneration resulting in loss of vision. In typical PRA rod photoreceptor responses are lost first followed by cone photoreceptor responses [5]. Fundus changes observed in PRA are bilateral and symmetrical and include tapetal hyper-reflectivity in the early stages followed by vascular attenuation, pigmetary changes and atrophy of the optic nerve head in the later stages of disease (Figure S1) [6]. Numerous forms of PRA have been documented in more than 100 dog breeds and while they exhibit similar clinical signs, the aetiology, age of onset and rate of progression vary between and within breeds. Several disease-causing genes have been reported for some forms of PRA [7], [8], [9], [10], [11], [12], [13], [14], [15], [16], but many remain undefined.

Canine diseases have already proved valuable natural models for the study of many varied human conditions such as cardiac conotruncal malformations [17], myotubular myopathy [18] and hereditary retinopathies such as Leber congenital amaurosis and achromatopsia [15], [19], [20]. Further to this, canine models for human eye diseases have proved invaluable in gene-therapy studies, most notably the canine model of Leber congenital amaurosis associated with *RPE65* [21], [22].

Most PRA cases in the Golden Retriever (GR) are clinically indistinguishable from other forms of PRA. The mode of inheritance appears from pedigree information to be autosomal recessive. While the age of diagnosis is most commonly at a relatively late age of approximately 6 years there is a great deal of variation within the breed. In the closely related Labrador Retriever breed, the only known form of PRA is called progressive rod cone degeneration (*prcd-*PRA) [8], a form of PRA that affects at least 22 breeds. To date only the *prcd*-PRA mutation has been associated with PRA in the GR. However, only a small number of PRA-affected golden retrievers have been found to be homozygous for the *prcd*-mutation. Worldwide the GRs for which PRA can be explained by the mutation in the *prcd* gene are in the minority [23].

Here we report the identification of a single base insertion mutation in *SLC4A3,* a gene encoding a solute carrier protein that is expressed in the retina. The mutation causes a shift in the reading frame resulting in a subsequent premature termination codon. We present evidence that this mutation represents a major susceptibility locus for late onset PRA, known hereafter as GR_PRA1, in Golden Retrievers.

## Results

### *Prcd*-PRA screening

All dogs displaying clinical signs typical of PRA, including a hyper-reflective tapetum and attenuated blood vessels, were diagnosed as affected with PRA (Figure S1).

To exclude the possibility that the affected GRs were positive for the mutation already known to cause *prcd*-PRA, all 80 of our GR cases were investigated for the previously described, autosomal recessive *prcd*-PRA mutation [8]. None of the affected GRs tested in this study was found to be homozygous for the *prcd*-PRA mutation. A single individual was heterozygous (G/A) while the remaining 79 were homozygous for the wildtype (normal) allele (G/G).

### Genome–wide Association Mapping

**Figure 1:**
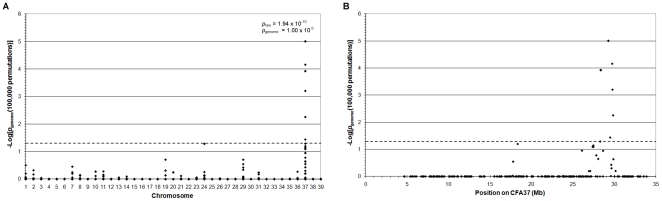
Genome-wide association mapping of PRA in Golden Retrievers. −Log_10_ of p-values after correction for multiple testing and population stratification with 100 000 permutations and IBS clustering, respectively. The dashed lines indicate the 5% significance level. **A**) −Log_10_ plot of genome-wide association results show a strong statistical signal on CFA37 (P_raw_ = 1.94×10^−10^, P_genome_ = 1.00×10^−5^). CFA39 represents the X chromosome. The most significant of the raw and permuted values are indicated. **B**) The statistically associated SNPs on CFA37 span 1.6 Mb from 28.331 Mb to 29.847 Mb.

**Figure 2:**
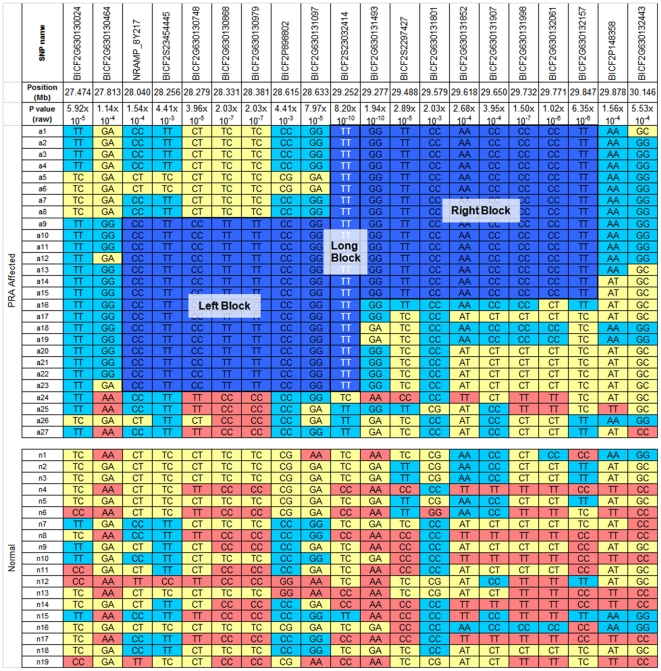
Fine mapping using haplotype analysis. SNP genotypes for 27 PRA cases and 19 PRA controls, over the 1.6 Mb region identified during the GWA study. Fifteen of the cases share a homozygous haplotype of eight SNPS (Left Block) and 15 cases share another homozygous haplotype of nine SNPS (Right Block). These two homozygous blocks overlap by just one SNP (Shared Block), defining the critical region of 644 kb between 28.633 Mb and 29.277 Mb on CFA37, a region containing 27 genes. Four of the cases do not share any haplotypes with the other 23 cases, but do share haplotypes with the controls.

Genome-wide association analysis of genotyping data from 46 GR dogs, 27 cases and 19 controls over the age of 8 when last examined, genotyped with 14,389 SNPs revealed a genome-wide significant association on chromosome 37 (CFA37; P_raw_ = 1.14×10^−11^, P_genome_ = 1.0×10^−5^). Some of the dogs used were known to be related through established pedigrees and therefore the presence of population stratification was expected. Identity-by-state (IBS) clustering in PLINK using genome-wide SNP marker data confirmed the presence of population stratification with a genomic inflation factor of 1.44. The signal on CFA37 remained significant (P_raw_ = 1.94×10^−10^, P_genome_ = 1.0×10^−5^) after correction for this by analysing for association within the IBS clusters and combining the resulting evidence with Cochran-Mantel-Haenszel (CMH) meta-analysis in PLINK [24] (Fig 1). The significantly associated region on CFA37, defined as the region encompassing significantly associated SNPs, extended from 28.331 to 29.847 Mb with the most significantly associated SNP (BICF2G630131493) at 29.277 Mb. In addition, a number of weaker signals were seen on other chromosomes including 19, 24 and 29, but only one marker on CFA24 reached near-significance (P_raw_ = 4.04×10^−5^, P_genome_ = 0.052). Using homozygosity mapping we identified two overlapping homozygous regions on CFA37 from 27.813 to 29.277 Mb and 28.633 to 29.878 Mb (Fig 2) and most of the PRA cases (23/27) analysed were homozygous for one or both of these regions. These homozygous regions overlap from 28.633 Mb to 29.277 Mb on CFA37 (Shared Block in Fig 2) and this 644 kb region was defined as the PRA critical region. It is likely the four dogs that do not carry the haplotype associated with the critical region are affected with a form of PRA that is genetically distinct from that addressed here.

### Candidate gene sequencing

**Figure 3:**
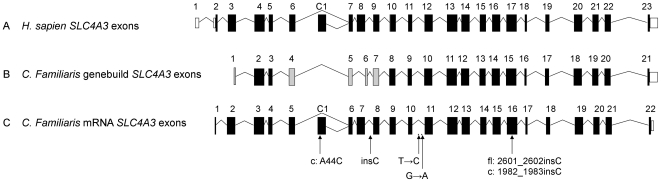
Graphical comparison of the exons and exon-intron boundaries of human and canine *SLC4A3.* A) Human (*Homo sapien*) *SLC4A3*. B) Canine (*Canis familiaris*) *SLC4A3* as predicted by Ensembl genebuild. Sixteen of the genebuild exons predicted are identical to the human exons (black). Exons 1, 4, 5 and 7 (grey) have differences in 5′ and/or 3′ exon-intron boundaries. Exon 6 (grey) shows no sequence similarity to its human equivalent and is probably incorrect. C) Canine *SLC4A3* exons confirmed by sequencing the retinal transcript. Exons 1, C1 and the correct exon 7 have not been predicted by Ensembl genebuild. The locations of the five fully-segregating sequence variants (in two PRA cases, two obligate carriers and four normal samples) are indicated, but only two are exonic and nonsynonymous. One of these is in the first exon of the cardiac isoform only (c: A44C) while the other is found in both the cardiac (c: 1981_1982insC) and full-length (fl: 2601_2602insC) isoforms of *SLC4A3*.

**Figure 4:**
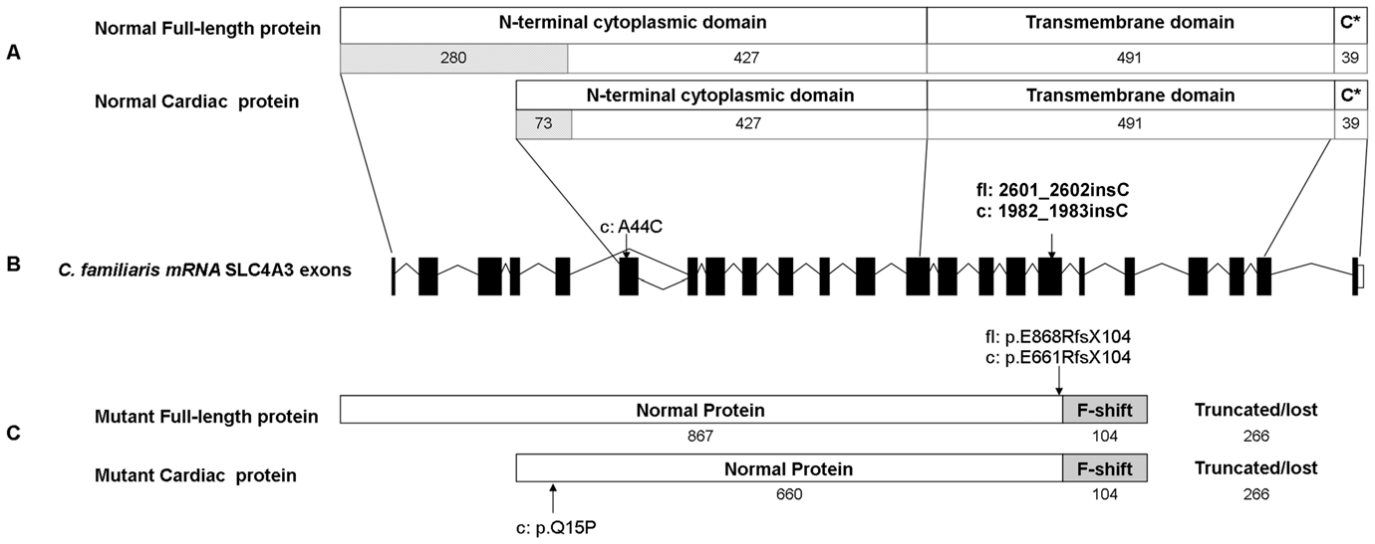
Graphical representation of the *SLC4A3* protein. A) Both isoforms are made up of a transmembrane domain flanked on both sides by a cytoplasmic domain (represented by C*). The number of amino acids that make up each domain are indicated. Several amino acids at the N-terminus are unique to either the full-length (280 residues) or cardiac (73 residues) isoform, but the remainder of the protein is identical in both isoforms. B) The locations of the two nonsynonymous SNPs in the confirmed canine *SLC4A3* transcript. C) The cardiac isoform alone is affected by the A44C SNP mutation (p.Q15P). Both isoforms are affected by the 2601_2602insC mutation. 867 amino acids at the N-terminus of the full-length protein and 660 of the cardiac isoform are normal. However the insertion causes a shift in the reading frame of 104 amino acids, leading to a premature termination codon. This results in a truncated protein product, lacking 266 residues of the C-terminus of both isoforms, if expressed. Therefore a large part of the transmembrane region and the entire c-terminal cytoplasmic region is absent in the mutant proteins.

There are 27 genes within the PRA critical region (Table S3), of which 25 have orthologous genes in the human and mouse. *NHEJ1* has been associated with Collie Eye Anomaly, an ocular eye condition clinically distinct from PRA. None of the remaining 26 these genes have previously been associated with ocular function or retinal degeneration in humans, however a solute carrier anion exchanger gene (*SLC4A3*) located within this region was identified as a strong candidate based on function and its association with retinal degeneration in mice [25], [26], [27]. Alignment of canine, human and mouse genomic and coding sequences revealed several inconsistencies and possible errors in the prediction of exon-intron boundaries of canine *SLC4A3* (Fig 3), including missing exons that are probably due to gaps in the reference sequence, perhaps due to extremely high GC content in these regions. Primers were designed to ensure sequencing coverage of all possible exons. To identify possible causal mutations at this locus, we sequenced 20 of the 23 *SLC4A3* exons and splice sites in genomic DNA from six GRs (two PRA-affected dogs, two obligate carriers and two unaffected dogs). DNA from a Miniature Long-Haired Dachshund and a Border Collie, chosen randomly, was also sequenced as additional controls. The CanFam2.0 reference sequence and annotation for *SLC4A3* is incomplete and includes two gaps that cover exons 1, 2 & 7 of the gene (Fig 3C). All attempts at sequencing across the two gaps were unsuccessful. Comparison of our sequence data with the canine reference genome sequence revealed 50 sequence variants. Five of the sequence variants showed perfect segregation with PRA within the eight samples sequenced, and of these two are exonic (Fig 3C). An A to C transversion at position 44 (c.A44C; CFA37:29,141,439) resulting in a nonsynonymous substitution (p.Q15P) was found in the first exon of the cardiac specific isoform, but does not affect the full-length isoform also present in the eye (Fig 4). A comparison among 33 eutherian mammals (data not shown) showed that the position is not well conserved, indicating that the sequence variant may not be functionally important. The second exonic change was a frame shift mutation caused by the insertion of a single cytosine in exon 16 (c.2601_2602insC; CFA37:29,147,633). It is predicted to cause a premature stop codon in exon 18 (p.E868RfsX104) possibly resulting in degradation of the mRNA by nonsense-mediated decay (NMD) or a truncated protein product (Fig 4). Exon 16 is included in both the cardiac specific isoform and the full-length isoform of *SLC4A3* that are normally expressed in the retina [26]. While the GR breed is known to suffer from hereditary cardiac diseases such as Subvalvular Aortic Stenosis (SAS) and Cardiomyopathy there is no evidence to indicate that dogs affected with GR_PRA1 experience any cardiac problems.

### Transcript evaluation

All of the coding sequence of the *SLC4A3* retinal transcripts, from both the main isoforms (SLC4A3_fl_ and SLC4A3_c_), was successfully sequenced in a healthy dog revealing that both isoforms are transcribed in the canine retina. In addition, our results show intron-exon boundaries identical to those of the human (Fig 3), which is in conflict with the boundaries predicted by Ensembl genebuild for the canine gene.

Alternative splicing of exon 5 of the full-length isoform results in two “full-length” isoforms that differ by the presence (*SLC4A3*_fl1_) or absence (*SLC4A3*_fl2_) of 27 nucleotides at the 5′ end. We were unable to sequence the full 5′ and 3′ UTRs for any isoform. From the sequencing of the retinal mRNA transcripts of all three isoforms we discovered that canine *SLC4A3*_fl1_ (Genbank accession no HQ379706) contains 1237 amino acids, *SLC4A3*_fl2_ (Genbank accession no HQ379707) contains 1228 amino acids and *SLC4A3*_c_ (Genbank accession no HQ379708) contains 1030 amino acids, with molecular weights of 136 kDa, 135DA and 114 kDa respectively, predicted using the ExPASy Proteomics Server [28].

### Mutation screening

**Figure 5:**
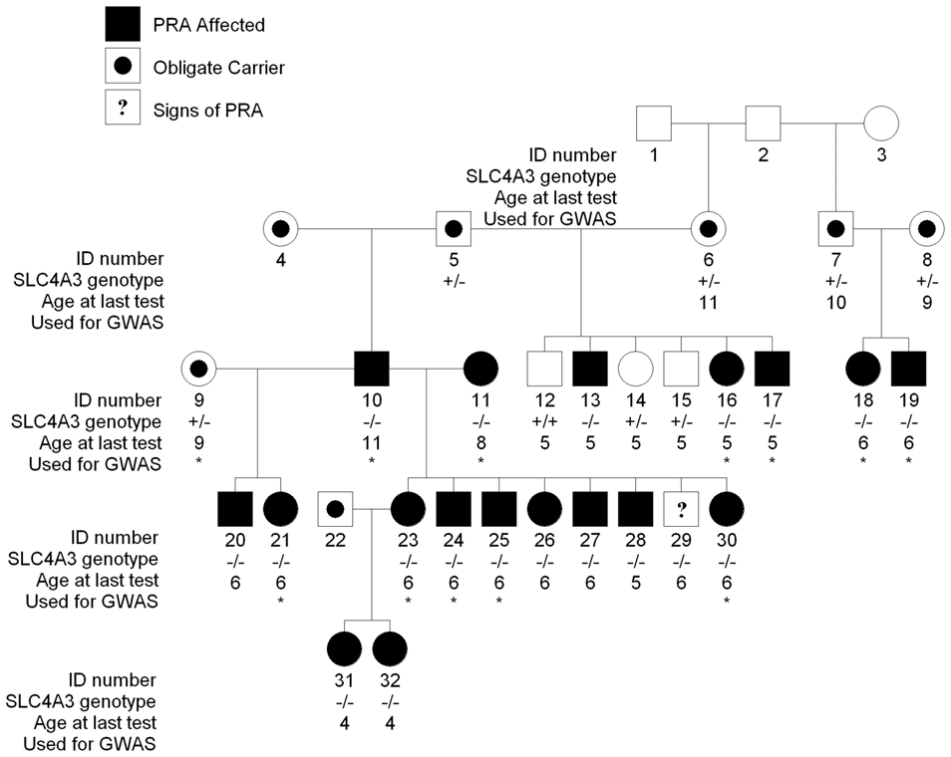
Segregation of GR-PRA1 in a family. GR-PRA1 appears to be recessive and fully penetrant, with the exception of one dog not yet diagnosed. Individual 29 is not clinically affected with PRA, but did show ophthalmascopic signs of the disease at the age of 6. Individuals 31 and 32 were only 4 years old when examined at the same time as a number of relatives including their mother, and were diagnosed with early-stage PRA. Individuals that formed part of the genome-wide association study are indicated with “*”.

We screened 48 GR dogs (21 cases and 27 controls present in the original GWA data set; Figure S2) for the two coding variants, A44C and 2601_2602insC, to investigate the association of both these variants with PRA and compare them with BICF2G630131493. The genotypes of the two variants i.e. homozygous wild-type, heterozygous and homozygous for the rare variant were concordant in all but six dogs (Figure S3), indicating the two variants are in very close linkage disequilibrium (r^2^ = 0.9118). Both variants, A44C and 2601_2602insC, showed significant allelic association with PRA (p = 3.4×10^−6^ and 7.5×10^−6^ respectively) although they were both less strongly associated than BICF2G630131493 (p = 6.9×10^−7^). However, under a recessive model we determined that 2601_2602insC was the most strongly associated (p = 7.9×10^−7^) when compared with A44C or BICF2G630131493 (p = 0.016 and p = 0.001 respectively). Neither variant shows complete association with PRA, but 2601_2602insC has a stronger likelihood of a deleterious effect on the protein. In addition, neither the nucleotide nor the amino acid affected by A44C is conserved in 33 eutherian mammals (data not shown). We therefore discarded this variant from further analysis. Analysis of the segregation of 2601_2602insC with PRA in a family of Swedish ancestry (Fig 5) indicates that GR_PRA1 is recessive and fully penetrant.

We screened 897 additional GR dogs making a total of 945 GRs tested for the 2601_2602insC variant (Table 1) to confirm that the mutation is not a commonly occurring polymorphism in this breed. 56.3% of known PRA cases were homozygous for the 2601_2602insC mutation (*SLC4A3* ^−/−^) and 87.2% of obligate carriers were heterozygous (*SLC4A3* ^+/−^). 99.7% of dogs known to be clinically free of PRA were either carriers of the mutant allele (10.4%) or homozygous for the wildtype allele (89.3%; *SLC4A3* ^+/+^). These GRs are from a number of different countries including known PRA cases from the UK, Sweden, France and the USA (Table 2). The mutation described here accounts for the majority of PRA cases from the UK and Sweden, 60.0% and 76.0% respectively. However it accounts for only a minority of PRA cases from France and Finland, 12.5% and 33.3% respectively, and none of the cases from the USA. A subset of the complete sample cohort, 108 from the UK, 109 from Sweden, 89 from France and 148 from the USA, were used to calculate allele frequencies of 0.04, 0.06, 0.02 and 0.00 respectively.

**Table 1:** GR_PRA1 genotypes and PRA clinical status for 945 GRs.

	PRA Clinical Status				
Genotype^i^	PRA Case	PRA Carrier	Clear	Unknown	Total
**SLC4A3^−/−^**	45 (56.3%)	1 (2.5%)	1 (0.3%)	1 (0.2%)	48
**SLC4A3^+/−^**	8 (10.0%)	34 (87.2%)	39 (10.5%)	28 (6.1%)	109
**SLC4A3^+/+^**	27 (33.7%)	4 (10.3%)	329 (89.2%)	428 (93.7%)	788
**Total**	80	39	369	457	945

i The wildtype allele is represented by “+” and the mutant allele by “−”.

**Table 2:** PRA cases grouped by country of origin and GR_PRA1 genotype.^i^

Genotype	UK	Sweden	France	Finland	Canada	USA	Total
**SLC4A3^−/−^**	5 (55.6%)	38 (76.0%)	1 (12.5%)	1 (33.3%)	0 (0.0%)	0 (0.0%)	45 (56.3%)
**SLC4A3^+/−^**	1 (11.1%)	4 (8%)	0 (0.0%)	0 (0.0%)	3 (100.0%)	0 (0.0%)	8 (10.0%)
**SLC4A3^+/+^**	3 (33.3%)	8 (16.0%)	7 (87.5%)	2 (66.7%)	0 (0.0%)	7 (100.0%)	27 (33.7%)
**Total**	9	50	8	3	3	7	

i All of the cases are free of *prcd*-PRA.

To determine whether or not 2601_2602insC is causal for PRA in other breeds we screened 48 dogs from four non-GR breeds made up of PRA cases of unknown aetiology and unaffected dogs: 10 Gordon Setter (seven cases), 10 Irish Setter (5 cases), 10 Tibetan Spaniels (eight cases) and 18 English Springer Spaniel (nine cases). All 48 dogs were homozygous wild-type (*SLC4A3* ^+/+^).

Finally, to determine whether 2601_2602insC is present in healthy dogs of other breeds we screened a further 223 dogs from three closely related breeds most likely to share polymorphisms with the GR (49 Chesapeake bay retrievers, 82 Labrador retrievers and 92 Flatcoat retrievers). All 223 dogs were homozygous wild-type (*SLC4A3* ^+/+^).

## Discussion

Several forms of PRA are present in the GR, including the *prcd* variant, but this mutation does not account for the majority of cases, particularly in Europe. Using a genome-wide association (GWA) analysis approach, we have identified a novel candidate variant in the *SLC4A3* gene that is likely to represent a major susceptibility locus for PRA in the GR. We found that while this mutation does not explain all cases of PRA in our study, suggesting that there are additional loci causing PRA in this breed, it does appear to be fully penetrant and a common cause of PRA in the breed. In our study we tested 80 GRs with PRA for the *prcd* mutation and found that only one dog was a carrier. Our findings therefore indicate that PRA is caused by at least three mutations in this breed.

*SLC4A3* was identified as a strong candidate causal locus within the PRA-critical region as it mediates Cl^−^/HCO_3_^−^ exchange across cellular membranes [27] and is expressed in various tissues including the Müller and horizontal cells of the retina [26]. Interestingly, the effect of *SLC4A3*-deficiency has been described in a knockout mouse model showing that a selective inner retina defect is followed by photoreceptor degeneration [25], similar to the retinal degeneration seen in many forms of PRA and RP.

*SLC4A3* occurs in two main isoforms, *SLC4A3*_fl_ (full length) and *SLC4A3*_c_ (cardiac), which have identical transmembrane and C-terminal regions while the N-terminal amino acids differ (Fig 4A) [26]. *SLC4A3*_fl_ is expressed predominantly in the brain, but it is also found in the gut, kidney [29], heart [30] and Müller cells of the retina [26]. The cardiac isoform is expressed predominantly in the heart [30] and also in the horizontal neurons of the retina [26].

The two exonic and nonsynonymous variants (c.A44C and c.2601_2602insC) are in tight linkage disequilibrium (r^2^ = 0.9118) for the 48 dogs screened for both mutations. In six dogs (three cases, one obligate carrier and two unaffecteds) however, variant alleles are not inherited together. Under an allelic model BICF2G630131493 is more strongly associated with PRA than either of the variants due to the heterozygous state of more of the cases than either of the variants. However, PRA in the GR appears to be inherited in an autosomal recessive manner and under a recessive model the 2601_2602insC variant is most strongly associated with PRA and it appears to be fully penetrant. Interestingly, this variant along with *prcd* still does not account for all cases of PRA in the GR indicating the presence of at least one additional mutation for PRA in the breed that remains to be identified.

Sequencing of *SLC4A3* from healthy retinal mRNA served three purposes. Firstly it confirmed the presence of “cardiac” and “full-length” mRNA transcripts (*SLC4A3_c_* and *SLC4A3_fl_* respectively) in the normal canine retina. Secondly it revealed that the exon-intron boundaries predicted by genebuild for the dog are incorrect for five of the first eight exons. They are instead identical to the human and mouse boundaries. Thirdly it revealed exons orthologous to part or all of human exons 1, 2, 3 and 8, that are absent from the Ensembl canine predictions (Fig 3A). Two large gaps in the reference sequence are found upstream of canine exons 2 and 8, and it is very likely that the missing exons lie within these gaps (Fig 3C). As is the case in humans and mice, canine *SLC4A3*_fl_ is alternatively spliced to produce two “full length” isoforms (*SLC4A3*_fl1_ and *SLC4A3*_fl2_). The functional implication of the difference between these isoforms is currently unknown.

In order to further test the validity of the insertion mutation, we screened 945 Golden Retrievers for the mutation (Table 1). We found that 56.3% of the PRA cases, 87.2% of the obligate PRA carriers and 99.7% of clinically unaffected dogs (which could be clear of the mutation or carry a single copy) have *SLC4A3* genotypes that are concordant with their clinical status. There are two groups of dogs with genotypes discordant with their phenotypes. The first comprises three PRA-clear dogs that are *SLC4A3* ^−/−^ (one obligate carrier, one PRA-clear and one not diagnosed as affected, but on re-examination is showing early signs of PRA). All three dogs were examined by a veterinary ophthalmologist at 4–7 years of age and as average age of onset is approximately seven years they would not necessarily have been displaying clinical signs at the time of their examination. Thus 93.8% of dogs (45/48) homozygous for the mutation i.e. *SLC4A3* ^−/−^, have developed PRA, suggesting that the mutation is fully penetrant, or nearly so. The inheritance observed in a pedigree of 32 dogs (18 cases) is supportive of a recessive mode (Fig 5). The second group comprises 35 PRA-affected dogs that are not *SLC4A3* ^−/−^ and four obligate carriers do not carry SLC4A3_2601_2602insC_. It is formally possible that the mutation has a dominant mode of inheritance with incomplete penetrance, or complex trait or compound heterozygote effects. However, we are pursuing these discordant cases in a separate investigation, and preliminary results indicate that a locus other than *SLC4A3* or *prcd* may be associated with PRA in these dogs (unpublished).

The absence of the mutant *SLC4A3* allele from non-GR breeds tested, including some dogs affected with PRA, and from GRs of US ancestry indicates that the mutation is rare and probably confined to GR lines with European ancestry. The mutant allele frequency of approximately 4% indicates that 1 in 576 GRs in the UK is affected with this form of PRA. However this is likely an over-estimate as a sample cohort collected for research is unlikely to be representative of the UK GR population. The allele frequency of approximately 6% in Sweden and 2% in France indicate 1 in 280 GRs of Swedish ancestry and 1 in 2000 of French ancestry are affected with this form of PRA. While it is always difficult to predict allele frequencies with accuracy these cohorts are probably more representative as the samples were not recruited for any particular study. Although 2601_2602insC has not been found in any GRs from the USA, including PRA cases, we cannot rule out the possibility that GR_PRA1 may be present in the US GR population as it is such a numerically large breed and the number of cases we tested is very small. In addition, three of the PRA cases that carry GR_PRA1 are Canadian dogs, indicating the disease allele is present in North America.

The Solute carrier family 4, anion exchanger, member 3 (SLC4A3, aka AE3) protein is part of a group of Na^+^-independent anion exchangers and it exchanges Cl^−^ for HCO_3_^−^ [31]. Little is known about the structure of the protein, but it is thought to be similar to family member *SLC4A1*. SLC4 proteins are made up of three structural domains (Fig 4A). At the N-terminus there is a hydrophilic, cytoplasmic domain of between 400 and 700 amino acids, followed by a hydrophobic, polytopic transmembrane domain of approximately 500 amino acids and lastly a cytoplasmic domain of between 30 and 100 amino acids at the C-terminal end [29].

The mutation, 2601_2602insC (p.E868RfsX104) in *SLC4A3*fl and 1981_1982insC (p.E661RfsX104) in *SLC4A3*c, occurs in the transmembrane region and as a result is likely to affect both full length and cardiac isoforms of the protein (Fig 4B and 4C). The shift in the reading frame results in the incorrect sequence of 104 amino acids and the loss of 266 residues at the C-terminal end of the protein. These residues constitute a large portion of the transmembrane region and the complete C-terminal cytoplasmic region. We have been unable to obtain retinal tissue from a dog affected with PRA and can therefore not confirm whether the protein is expressed in a truncated form or not expressed at all due to nonsense-mediated decay of the mRNA transcript. In the event of truncated protein expression functionally important regions or residues are lost. Glycosylphosphatidylinositol-linked carbonic anhydrase IV [32] and transmembrane carbonic anhydrase IX [33] interact with exofacial portions of the transmembrane domains and the C-terminal tail of the SLC4 polypeptides contains acidic motifs that may serve as cytoplasmic carbonic anhydrase II (CAII) [34], [35] binding sites, all of which affect HCO_3_^−^ homeostasis in the retina. Dahl *et al* found that a deletion of the C-terminal tail resulting in a lack of the CAII-binding site led to a loss of Cl^−^/HCO_3_^−^ exchange [36]. A number of possible functions for *SLC4A3*_fl_ in Müller cells have been proposed including: 1) Intracellular CO_2_ released by photoreceptors is converted into HCO_3_^−^ and H+ by carbonic anhydrases. It is thought that removal of these products into the blood or vitreous is facilitated by transporters such as *SLC4A3* in the Müller cell end feet [37]. 2) *SLC4A3*_fl_ may facilitate the exchange of intracellular HCO_3_^−^ for extracellular Cl^−^, thereby contributing to pH maintenance [26] and a lack of bicarbonate in the retina results in loss of retinal function [38]. 3) Finally it may play a role in Cl^−^ level maintenance which potentially affects Cl^−^-dependent transporter activity e.g. transporters of γ-Aminobutyric acid (GABA) and taurine [39]. Horizontal cells also express various carbonic anhydrases which could in turn create significant levels of intracellular HCO_3_^−^. By exchanging this HCO3^−^ with extracellular Cl^−^, *SLC4A3* may contribute to pH_i_ homeostasis [26].

PRA caused by the mutation described here has an average age at diagnosis of 6.64 years and this is indicative of a late age of onset (data not shown). The two affected individuals that were diagnosed at the relatively young age of 4 years (Individuals 31 and 32 in Fig 5) were examined at the same time as a number of relatives including their mother. In these two dogs the disease was in the early stages. The late age at diagnosis corresponds to the late-onset findings of Alvarez et al where *SLC4A3* knockout mice appeared completely normal at 4 months of age [25]. However on closer investigation the mice displayed an inner retina defect from birth, which led to a failure of phototransduction at 4 months, followed by pathological signs of photoreceptor degeneration at 8 months and eventually complete blindness. Pathological signs included defects in retinal blood vessels, the optic nerve head and rod bipolar cells [25], consistent with most forms of PRA. *SLC4A3* deficiency also resulted in increased expression of CAII and CAXIV enzymes as well as the Na^+^/HCO_3_^−^ co-transporter, NBC1, in the horizontal and Müller cells. The authors hypothesised that while these changes possibly compensated for the loss of *SLC4A3* to an extent, the ability of the cells to maintain acid-base balance is still compromised, leading to late-onset photoreceptor cell death [25]. The discordant GR PRA cases i.e. *SLC4A3* ^+^/^+^ and *SLC4A3* ^+/−^ tended to develop PRA at an earlier age, with an average age at diagnosis of 5.27 years (data not shown), which is consistent with the segregation of a third form of PRA in the GR breed. It is becoming increasingly apparent that even in breeds with small effective population sizes, Mendelian conditions may be genetically heterogeneous. For example day blindness in the Alaskan Malamute breed is caused by two or more mutations, only one of which is currently known [40].

While PRA is widely considered to be the veterinary equivalent of RP, the limited characterisation of many forms of PRA at a cellular level suggests it may also be the equivalent of other retinal degenerations such as cone and cone-rod dystrophy. Further investigations are required to understand the cellular processes involved in this form of PRA, including whether the rod or cone photoreceptor cells are affected first. Alvarez et al identified *SLC4A3* as a candidate for hereditary vitreoretinal degenerations (HVD) in humans based on their work on a knockout mouse [25]. Therefore PRA caused by the 2601_2602insC mutation should be considered a potential model for all forms of human retinal degeneration including Leber congenital amaurosis (LCA) or cone-rod degeneration. Apart from the knockout mouse and the mutation described here no other mutations in this gene have been reported to be associated with retinal degenerations in any other species.

The identification of a frameshift insertion in *SLC4A3* in GR dogs with PRA, that is likely to be a major susceptibility locus for PRA in this breed, endorses the status of this gene as a candidate for human retinal degenerations. In addition, this novel form of PRA in the GR may prove to be a valuable model for further studies to enhance our understanding of visual pathways and gene therapy investigations.

## Materials and Methods

### Sample processing

All DNA samples were collected from privately owned pet dogs with the owners' consent. Sampling of blood from all Swedish dogs was performed by the Department of Animal Breeding and Genetics (SLU) according to ethical approval by the Swedish Board of Agriculture (C138/6), and from US dogs by the Broad Institute based on a protocol approved by the MIT CAC (*0907-068-10 and 1109-127-12). All other DNA samples were collected as buccal swabs. Blood samples were collected into EDTA tubes and genomic DNA was either extracted manually from peripheral blood leukocytes using QIAamp DNA Blood Midi Kit (Qiagen, Hilden, Germany) or automatically on a QIAsymphony SP/AS instrument (Qiagen, Hilden, Germany). DNA was also extracted from whole blood using a Nucleon Genomic DNA Extraction Kit (Tepnel Life Sciences, Manchester, UK), according to the manufacturer's instructions. For samples collected as buccal mouth swabs, DNA was extracted using a QIAamp® DNA Blood Midi Kit (Qiagen, West Sussex, UK). A retinal tissue sample from a dog of unknown breed that was free of PRA was taken post mortem, with the owner's consent. RNA was extracted using an RNeasy Protect Mini Kit (Qiagen, West Sussex, UK) according to the manufacturer's instructions.

The diagnosis of individual dogs was determined by veterinary ophthalmologists through the BVA/KC/ISDS (British Veterinary Association/Kennel Club/International Sheep Dog Society) Eye Scheme in the UK or the Swedish Kennel Club Eye Scheme in Sweden. Cases were defined as dogs diagnosed as affected with PRA on the basis that they displayed ophthalmoscopic signs consistent with PRA including tapetal hyperreflectivity and vascular attenuation. Controls were dogs free of inherited eye disease of any kind, and at least 8 years old at the time of examination for the genome wide association analysis or any age for subsequent investigations. Blood and buccal cheek samples that were used for the allele frequency investigations were donated by owners or collaborators, were all collected under the same conditions and with the same approval as described above and were unrelated at the parent level.

### *Prcd*-PRA screening

We genotyped DNA from 80 PRA-affected GRs for the *prcd* mutation using the TaqMan allelic discrimination technique (Applied Biosystems Inc., Foster City, CA) according to the manufacturer's instructions. Primers (Forward: 5′-GGCCTTTCTCCTGCAGACT-3′; Reverse: 5′-CAGCTTCTCACGGTTGGAC-3′) and PrimeTime Dual-Labelled Probes (G-probe: 5′-FAM-AGCCATGTGCACCACCCTCT-BHQ-3′ and C-probe: 5′-HEX-TGAGCCATGTACACCACCCTCT-BHQ-3′; IDT, Glasgow, UK) were designed with Primer3 [41]. PCR amplification and allelic discrimination plate read and analysis were carried out on a Techne Quantica Real Time Thermal Cycler with the Quansoft software (Bibby Scientific Limited, Staffordshire, UK).

### Genome-wide association mapping

CanineSNP20 BeadChips (Illumina Inc., San Diego, California, USA) were used to obtain genotype calls for 22,362 SNPs using DNA from 27 Swedish PRA cases and 19 Swedish controls. A genome-wide association (GWA) analysis was conducted using the software package PLINK [24]. SNPs with a minor allele frequency <5% and missing genotype calls >10% were removed from the analysis, resulting in a final data set of 14,389 markers. Sample call rate was >97% for all samples. Identity-by-state (IBS) clustering with PLINK was used to examine and adjust for population stratification. As a correction for multiple testing, we repeated the GWA analysis using the Max(T) permutation procedure in PLINK (100,000 permutations). Homozygosity mapping analysis was performed to narrow and define a critical region.

### Gene sequencing

To design primers for amplification and sequencing of the canine *SLC4A3* gene, a ClustalW [42] alignment was produced using the Ensembl predicted canine *SLC4A3* transcript (ENSCAFG00000015723) and available known mouse (ENSMUSG00000006576) and human (ENSG00000114923) Ensembl transcripts. Primers for amplification and sequencing of exons were designed with Primer3 [41] in the introns for amplification of genomic DNA (Table S1) and in exons for the amplification of cDNA (Table S2). Twenty of the 23 exons of *SLC4A3* were amplified by polymerase chain reaction using HotStarTaq Plus DNA Polymerase (Qiagen, West Sussex, UK) in genomic DNA from six GRs that were included in the GWA study (two cases, two obligate carriers and two controls) and DNA from one Miniature Long-Laired Dachshund and one Border Collie. Obligate carriers were dogs that are known to have produced PRA-affected offspring. *SLC4A3* mRNA sequence was amplified by reverse-transcriptase PCR using SuperScript®II Reverse Transcriptase (Invitrogen, Paisley, UK) according to the manufacturer's instructions. PCR products were purified using 96-well filter plates (Multiscreen HTS-PCR, Millipore, Billerica, MA). Amplification products were sequenced on an ABI 3100 DNA Analyzer using BigDye Terminator v3.1 (Applied Biosystems, Inc., [ABI], Foster City, CA). Sequence traces were assembled, analysed and compared using the Staden Package [43].

### Mutation screening

To further investigate two exonic, disease-segregating sequence variants in a larger dataset exon 16 of the full-length isoform and exon 1 of the cardiac isoform were sequenced in 48 GRs, made up of 21 cases, 9 obligate carriers and 18 GRs clear of PRA. These two exonic variants were analysed for association with PRA and compared with the most associated SNP marker, BICF2G630131493, under allelic and recessive models, using the software package PLINK [24]. The suggestive causative mutation for GR_PRA1 in exon 16 was screened in 945 GRs by PCR amplification using fluorescent primers (Forward: 5′-6-FAM-AGAGCAACCTTGTAACCCGTA-3′ and Reverse: 5′-GGAAGAAGGCAATGAGAAAGG-3′; IDT, Glasgow, UK) and subsequent fragment length polymorphism detection using an ABI 3100 DNA Analyzer and GeneMapper® Software (Applied Biosystems, Inc., [ABI], Foster City, CA). The panel of 945 GRs (including the 48 DNA samples already sequenced), was made up of 80 PRA cases, 39 obligate carriers and 826 clear dogs or dogs with unknown phenotypes of any age. In addition, samples from 48 dogs representing four other breeds that are known to be affected with different forms of PRA and from 223 dogs representing three breeds that are closely related to the GR breed were also included in the mutation screening.

## Supporting Information

Figure S1 **Fundus changes observed in typical PRA. A**) The fundus of a healthy Golden Retriever. **B**) The fundus of a Golden Retriever displaying signs typical of PRA in most breeds. The tapetum, which is the layer of cells behind the retina, appears hyper-reflective, the blood vessels are attenuated and the optic disc is pale. The photos were taken on the same day from littermates that were 6 years old.
(TIF)

Figure S2 **Use of samples in subsequent analyses for which genome wide SNP data is available.** 27 cases (blue) and 147 potential controls (grey) were genotyped on the SNP20 bead chip. The number of cases and controls used in each analysis is indicated.
(TIF)

Figure S3 **Concordance and association with PRA of variants (2601_2602insC and A44C) and SNP BICF2G630131493.** 35 cases are concordant at all 3 positions, 7 are concordant for the 2 variants and 6 are discordant. All 3 polymorphisms are significantly associated with PRA with a recessive mode of inheritance but 2601_2602insC far more so than A44C or BICF2G630131493.
(TIF)

Table S1 **Candidate gene primers.**
(XLS)

Table S2 **Candidate gene reverse transcriptase primers.**
(XLS)

Table S3 **Genes in the canine 644 kb PRA-critical region.**
(XLS)
