# Supplementary figures and images for: A Frameshift Mutation in Golden Retriever Dogs with Progressive Retinal Atrophy Endorses SLC4A3 as a Candidate Gene for Human Retinal Degenerations
Source: PLoS One. 2011 Jun 27;6(6):e21452. doi: 10.1371/journal.pone.0021452 (PMC3124514; doi:10.1371/journal.pone.0021452)

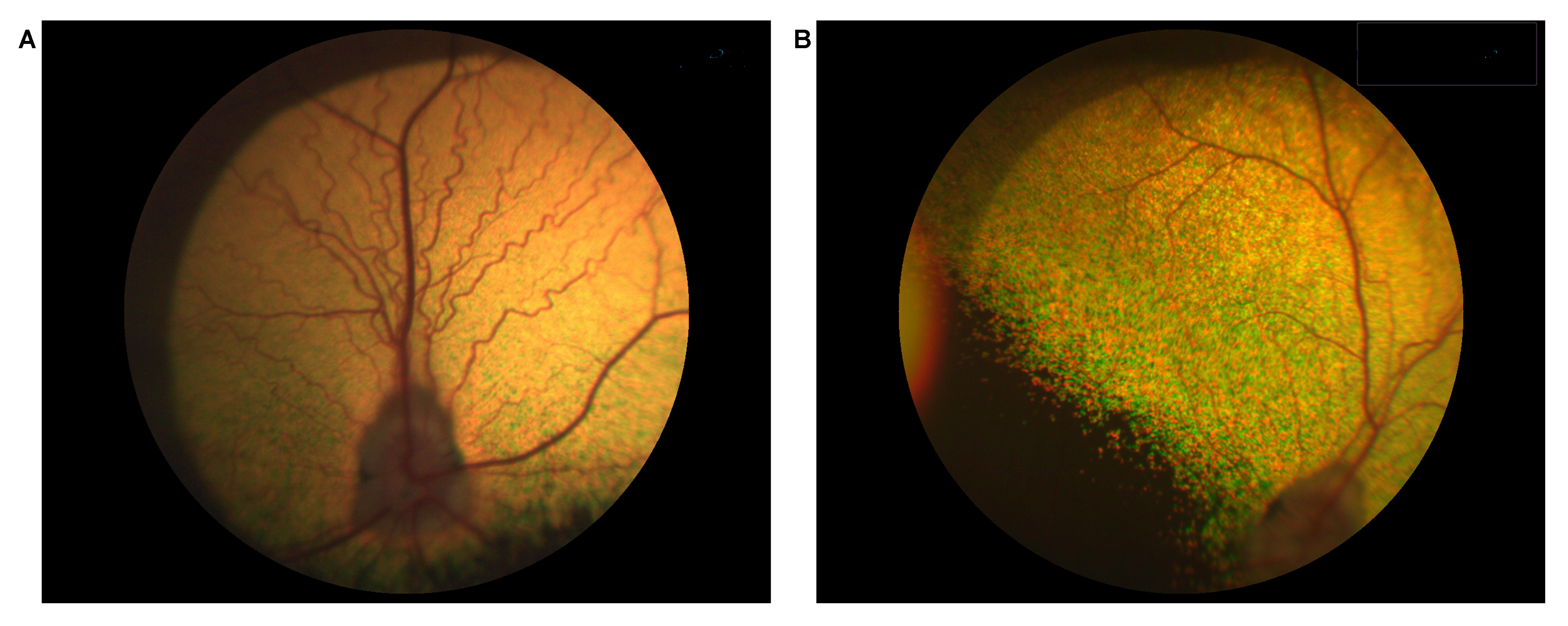

Supplement: Figure S1 — Fundus changes observed in typical PRA. A) The fundus of a healthy Golden Retriever. B) The fundus of a Golden Retriever displaying signs typical of PRA in most breeds. The tapetum, which is the layer of cells behind the retina, appears hyper-reflective, the blood vessels are attenuated and the optic disc is pale. The photos were taken on the same day from littermates that were 6 years old. (TIF) [file pone.0021452.s001.tif]

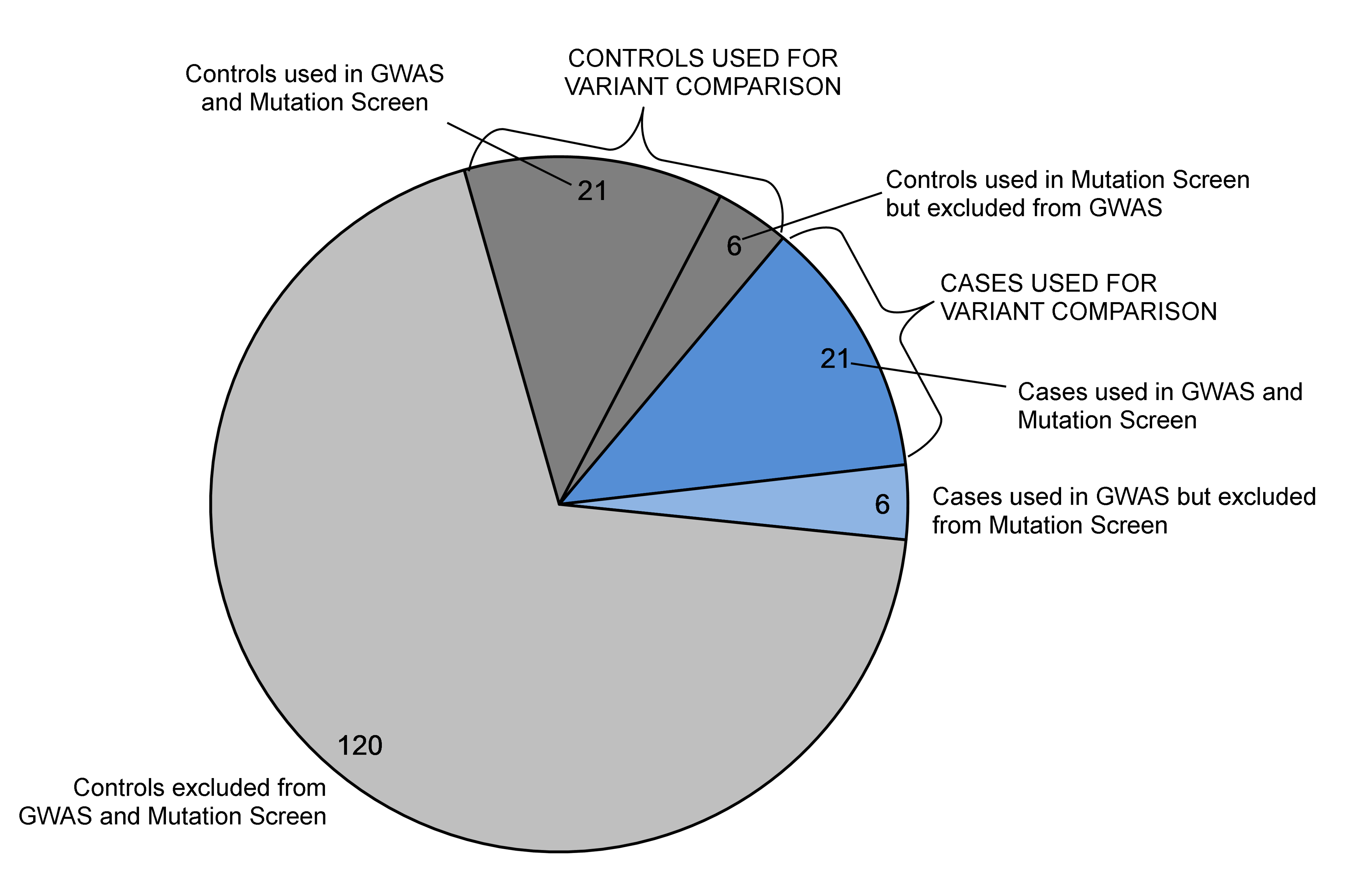

Supplement: Figure S2 — Use of samples in subsequent analyses for which genome wide SNP data is available. 27 cases (blue) and 147 potential controls (grey) were genotyped on the SNP20 bead chip. The number of cases and controls used in each analysis is indicated. (TIF) [file pone.0021452.s002.tif]

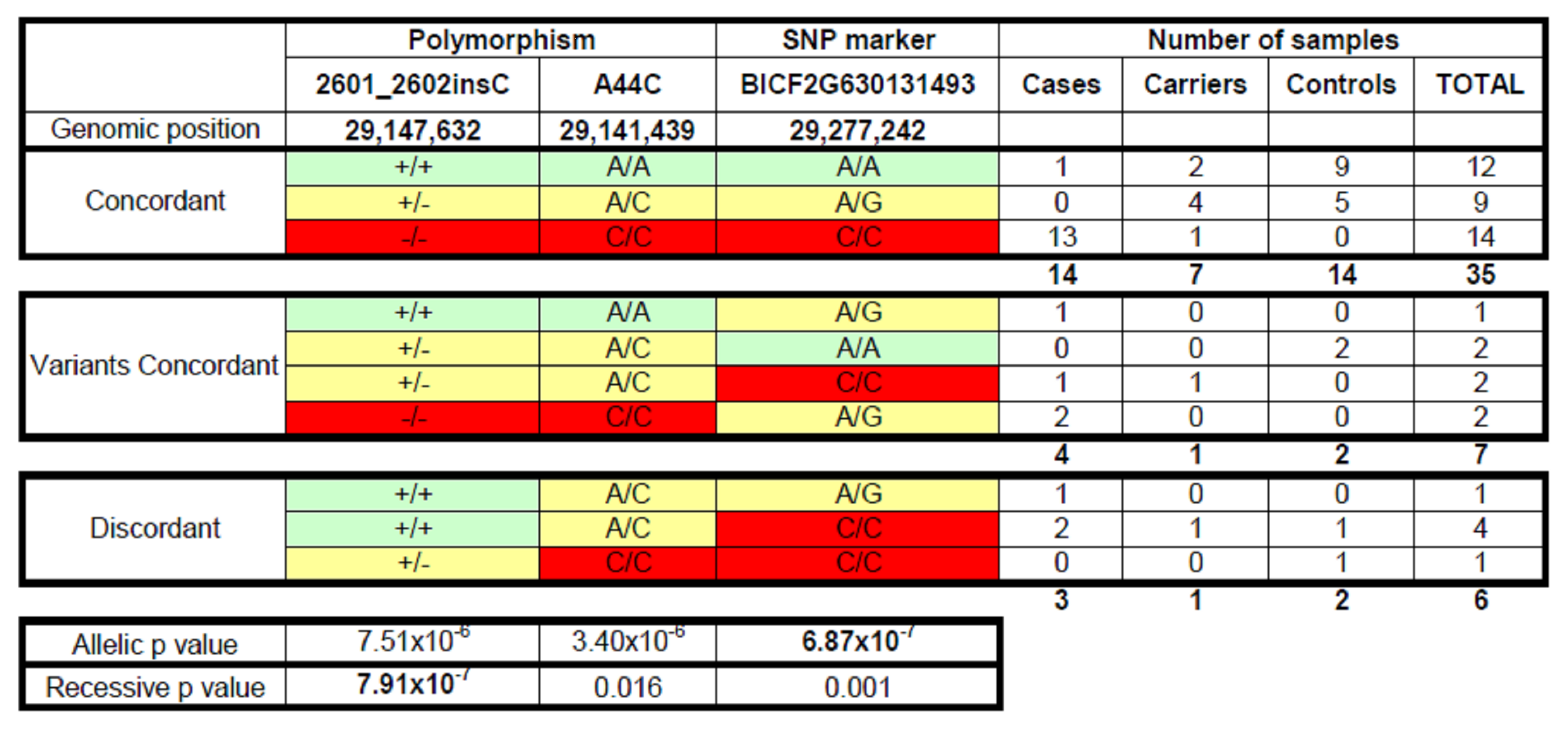

Supplement: Figure S3 — Concordance and association with PRA of variants (2601_2602insC and A44C) and SNP BICF2G630131493. 35 cases are concordant at all 3 positions, 7 are concordant for the 2 variants and 6 are discordant. All 3 polymorphisms are significantly associated with PRA with a recessive mode of inheritance but 2601_2602insC far more so than A44C or BICF2G630131493. (TIF) [file pone.0021452.s003.tif]
